# Conductance Ratios and Cellular Identity

**DOI:** 10.1371/journal.pcbi.1000838

**Published:** 2010-07-01

**Authors:** Amber E. Hudson, Astrid A. Prinz

**Affiliations:** 1 Bioengineering, Georgia Institute of Technology, Atlanta, Georgia, United States of America; 2 Biology, Emory University, Atlanta, Georgia, United States of America; École Normale Supérieure, College de France, CNRS, France

## Abstract

Recent experimental evidence suggests that coordinated expression of ion channels plays a role in constraining neuronal electrical activity. In particular, each neuronal cell type of the crustacean stomatogastric ganglion exhibits a unique set of positive linear correlations between ionic membrane conductances. These data suggest a causal relationship between expressed conductance correlations and features of cellular identity, namely electrical activity type. To test this idea, we used an existing database of conductance-based model neurons. We partitioned this database based on various measures of intrinsic activity, to approximate distinctions between biological cell types. We then tested individual conductance pairs for linear dependence to identify correlations. Contrary to experimental evidence, in which all conductance correlations are positive, 32% of correlations seen in this database were negative relationships. In addition, 80% of correlations seen here involved at least one calcium conductance, which have been difficult to measure experimentally. Similar to experimental results, each activity type investigated had a unique combination of correlated conductances. Finally, we found that populations of models that conform to a specific conductance correlation have a higher likelihood of exhibiting a particular feature of electrical activity. We conclude that regulating conductance ratios can support proper electrical activity of a wide range of cell types, particularly when the identity of the cell is well-defined by one or two features of its activity. Furthermore, we predict that previously unseen negative correlations and correlations involving calcium conductances are biologically plausible.

## Author Summary

Most motor neurons receive input from the brain before transmitting to the muscle, resulting in a muscle contraction. In some cases, a small group of motor neurons can act independently to control rhythmic muscle contractions. Locomotion in mammals is thought to arise, in a large part, due to neuronal networks of this type residing in the spinal cord. However, the cellular machinery that guarantees the needed rhythmic pattern of electrical activity in these neurons is not fully understood. Here, we use a small circuit that controls stomach contractions in crustaceans like crabs and lobsters, called the pyloric circuit, to investigate potential mechanisms for regulation of neuronal activity. Ion channel proteins are integral to determination of electrical activity type. Recently, experimental studies using cells of the pyloric circuit have shown correlations in the expression of these proteins. Our study uses a mathematical model of neuronal electrical activity to detail how these correlations may be influencing activity type. We found that correlations imposed on model parameters increase the likelihood of a desired behavior, and we therefore conclude that a biological cell utilizing ion-channel correlations will have the advantage of increased robustness of activity type.

## Introduction

In well studied neuronal networks, it is often observed that each neuron has a specific role in the function of the circuit. In some cases, this role is unique and vital, and the health of the animal depends on a robust cellular identity. One example of this occurs in the pyloric circuit of the crustacean stomatogastric ganglion (STG). This well-studied system must produce robust rhythmic activity for successful digestion [1], and does so through the dependable cellular properties of its component neurons. These neurons are identified by their reliable network activity, morphology, and connectivity [1]. This reliability is surprising, because identified cells can have different sets of ion channel maximal conductances in different animals of a population, despite generating the same predictable electrical activity [2], [3]. Furthermore, these ion channels are constantly replaced or modified due to synaptic input, sensory feedback, and neuromodulatory action [4], [5]. In light of these sources of variability, how stomatogastric cells produce consistent, stable output is an open question.

Recent experimental evidence suggests that coordinated expression and regulation of ion channels may play a role in constraining cellular electrical properties. The first such relationship found in the lobster STG was a positive co-regulation between the transient K^+^ current (I_A_) and the hyperpolarization-activated mixed ion current (I_h_) [6], [7]. The investigators discovered that cellular injection of mRNA coding for the ion channel underlying I_A_ caused an endogenous increase in the opposing I_h_ current, without changing the electrical activity of the cell. The same conductance pair was shown to be correlated in a population of identified neurons in the crab STG, and this correlation was found to be independent of the effects of neuromodulators [8]. The latter study compared correlations of current density in the pyloric dilator (PD) neuron before and after removal of top-down neuromodulatory input, and found two additional K^+^ conductance correlations that were neuromodulator-dependent. A variety of positive linear correlations in ion channel mRNA copy numbers have been found in other identified STG cell types, as well [9]. Each cell type appears to express a unique set of ion-channel correlation types and linear relationship slopes. In some cell types, correlations have been seen to involve three or four channel types. Similar relationships, involving conductances [10], [11], [12], or ion-channel kinetics [13], have been found in other model systems at the single cell (co-regulation) and population (correlation) levels. Combined, the data suggest a means for constraining cellular activity despite the often several-fold conductance variability seen in these cell types.

To date, all experimentally demonstrated conductance correlations within a wild type population of identified neurons have had a positive slope; no negative correlations have been found. This finding raises several questions about the mechanisms underlying the conductance correlations and their relation to maintenance or definition of activity type. At this time, the mechanisms are unknown, though there are several possibilities. Two membrane channel genes may be concurrently regulated by a single transcription factor in a cell-type specific manner [14]. Alternatively, a cleaved section of one ion channel can act as the transcription factor (or repressor) for another [15]. A unidirectional mechanism of this type may explain why the I_A_–I_H_ co-regulation is not reciprocal in STG neurons [16]. In addition, correlations may be controlled by coordinated post-transcriptional or -translational modification of one or more channel gene products [17], [18]. Though there is evidence for activity-independent co-regulation [6], [8], changes in activity are needed for co-regulation to be expressed during *Xenopus laevis* development [11]. This raises the possibility that activity-dependent regulatory cascades may also be involved in conductance co-regulation in some cases [19], [20]. A number of conductance correlations are also neuromodulator dependent [8]. There is a possibility that any of the above mechanisms are influenced by the presence of neuromodulator, and that correlation differences between cell types are due to differences in neuromodulator sensitivity [21], [22].

In addition to questions about the underlying mechanisms, questions remain regarding the cellular effects of linear conductance correlations. The effects on activity are likely different for each conductance pair; however, recent modeling studies have shed light on general trends in the structure of the conductance space with regard to activity type. Goldman et al. undertook a study of the pair-wise conductance space of stomatogastric neurons and neuron models, and demonstrated quasi-linear grouping of broadly categorized activity types [23]. Similar results have been obtained with a high-dimensional visualization technique of a model neuron database, wherein all conductance parameters are considered [24]. Hand-tuned models have also been a useful tool for demonstration of conductance correlation effects on activity. In one stomatogastric neuron model, a linear relationship between I_A_ and I_H_ maintains a specific number of spikes per burst [6]. Further theoretical studies demonstrated that correlated changes in these conductances can adjust neuronal input-output gain, while maintaining other features of activity [25]. Parameter changes in a leech heartbeat neuron model with narrowly constrained burst period and spike frequency demonstrated a correlation in the spike-mediated synaptic conductance and the inactivation time constant of the transient calcium current I_CaS_ [26]. A tempting conclusion to draw from these results would be that as more features of activity are constrained, more correlations may be required to maintain proper activity. This may not be true, as has been suggested previously [26]. Taylor et al. recently published a detailed model of the lateral pyloric (LP) neuron constrained to reproduce 9 experimentally measured activity characteristics, but found no strong linear correlations between conductance parameters [27].

Here, we expand on previous work by investigating the general relationship between the presence of conductance correlations and the manifest features of activity type. We hypothesize that conductance correlations do contribute to the robustness of critical features of electrical activity. To test this, we use a database of generic STG conductance-based model neurons [28]. These model neurons were first partitioned into groups based on ranges of a single characteristic of the electrical activity, or combinations of biologically-inspired activity characteristics. The conductance space for each group was examined and individual conductance pairs were tested for linear dependence. The conductance correlations found were further evaluated for their effect on activity type by building correlation-based populations of models. Our results show that while regulating conductance ratios can support the maintenance of a single cellular activity characteristic, the effects of correlations are less clear when multiple characteristics are required to specify activity type.

## Methods

### Model database

A model neuron database was used to investigate the relationship between conductance correlations and intrinsic activity type. This database has been previously described [28]. Briefly, a single-compartment conductance-based model was used. Seven of the eight conductance types used in this model were derived from experiments on unidentified stomatogastric cells in culture [29]. They include: a fast Na^+^ (g_Na_), fast transient Ca^2+^ (g_CaT_), slow transient Ca^2+^ (g_CaS_), fast transient K^+^ (g_A_), Ca^2+^-dependent K^+^ (g_KCa_), delayed-rectifier K^+^ (g_Kd_), and a voltage-independent leak conductance (g_leak_). The hyperpolarization-activated mixed-ion inward conductance (g_H_) was modeled after that found in guinea pig lateral geniculate relay neurons [30]. Each maximal conductance parameter was independently varied over 6 equidistant values, ranging from zero to a physiologically relevant maximum. By simulating the model neurons corresponding to all 6^8^ = 1,679,616 possible combinations of these parameters, a large variety of intrinsic electrical activity patterns were created. This systematic variation of parameters created an eight-dimensional grid of simulated parameter sets within the conductance space of the model. A single model neuron, with its particular intrinsic activity type, inhabits one grid point in this eight-dimensional space.

**Figure 1:**
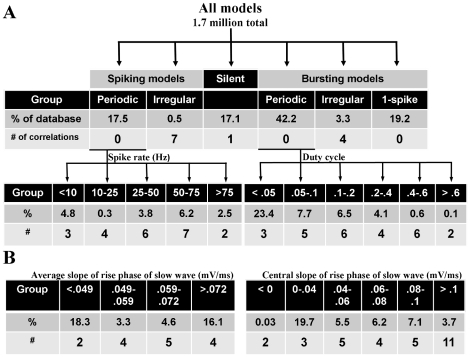
Levels of database segmentation based on single activity characteristics. (A) The first tier of this hierarchy includes all models in the database. These models are then divided into groups based on activity type. The second tier includes the groups periodic spiking, irregular spiking, silent, periodic bursting, irregular bursting, and one-spike bursting models. For each group, the number of models (shown in percent of database) and the number of correlations found within that group is shown. The groups “periodic spiking” and “periodic bursting” are further subdivided in the third tier. The spiking models are partitioned by spike frequency in Hz, and the bursting models are partitioned by duty cycle. (B) In addition to duty cycle, periodic bursting neurons were also partitioned based on the slope of the rising phase of their slow wave activity. Three measurement techniques and segmentation schemes were used, two are shown here. See methods.

**Figure 2:**
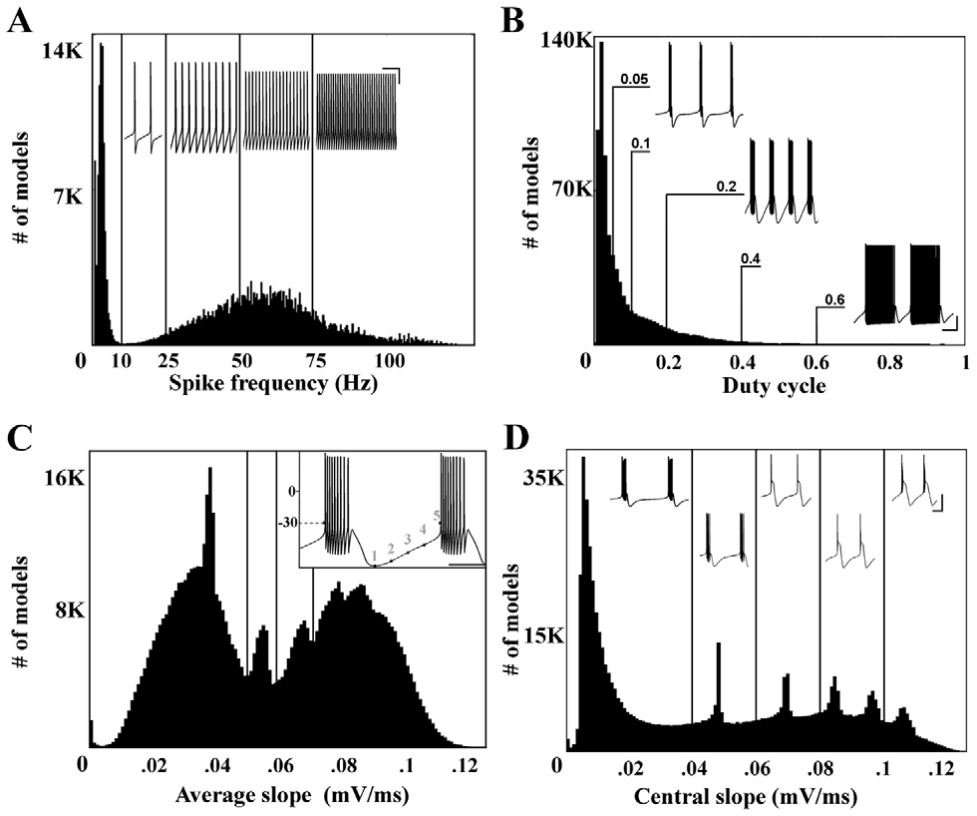
Histograms of main features used for partitioning the database. (A) Periodic intrinsically spiking model neurons sorted by spike frequency. Thin vertical lines indicate group boundaries used for sorting activity type. Voltage traces are for models at the lower bound of each group. Bin size is 0.5 Hz. Scale bars for voltage traces are 20 mV (vertical) and 100 msec (horizontal). (B) Periodic intrinsically bursting model neurons sorted by duty cycle. Labeled lines indicate group boundaries and the duty cycle of adjacent voltage traces. Bin size is 0.01. Scale bars for voltage traces are 20 mV and 500 msec. (C) Periodic bursting models were also sorted by the average slope of the rising phase of their slow wave activity. Inset shows the method of calculation of this slope. Average slope was calculated between points 1 and 4 (see inset), to avoid any artifact generated by the sharp incline of the first spike in the burst. Bin size 0.001 mV/ms. Scale bar for inset is 200 msec. (D) Periodic bursting models were finally sorted by the central slope of the rise phase of the slow wave. Central slope was calculated by taking the average slope between points 2 and 4 seen on the inset in part C. Bin size 0.001 mV/ms. Scale bars for voltage traces are 40 mV and 200 msec.

The currents used to generate this model were not based on any one STG cell type. To approximate distinctions between cell types, which are identified in part by activity, the database was partitioned based on intrinsic activity type of each model neuron. The first level of categorization divided the model neurons into five subsections: silent, periodic spiking, periodic bursting, irregular (non-periodic) spiking or irregular bursting (Figure 1). Silent models exhibited no membrane voltage maxima. Periodic spiking models had inter-maximum intervals that were consistent within 1% of their mean. Periodic bursting activity was detected and defined as follows. First, the peaks and troughs of the membrane potential were subjected to a spike detection threshold. Any peak greater than −30 mV was defined as a spike, and all others were ignored as sub-threshold activity. Next, the inter-burst interval was defined as any inter-spike interval that deviated from the length of the greatest inter-spike interval by less than 30%. These definitions are different from previous burst classification metrics [28] to avoid minor classification errors associated with alternating burst features. The second level of categorization further partitioned the periodic spiking and bursting models. The large group of periodic bursting neurons was either partitioned based on the duty cycle, defined as the fraction of the burst period taken up by the burst duration, or by the average slope between the inter-burst minimum and the start of the next burst, hereafter referred to as the rising phase of the slow wave (see Figure 2C). Briefly, the slope was calculated by first locating the inter-burst minimum (point 1 on the inset of Figure 2C). Then, the next crossing of the spike detection threshold (−30 mV) was recorded (point 5). The distance between the two points was divided into four equal sections (demarcated by points 2–4). Average slope was calculated between points 1 and 4, omitting the portion between points 4 and 5 to avoid any artifact generated by the sharp incline of the first spike in the burst. Initial slope (average slope between points 1 and 2) and central slope (average slope between points 2 and 4) were also investigated. These characteristics were chosen because both duty cycle and the average slope of the rise phase are thought to be regulated within a narrow range in biological cells [6], [31]. Periodic spiking neurons were similarly partitioned by spike frequency. Group size and boundaries were chosen based on population distributions shown in Figure 2. Clustering of models was seen in the spiking models (Figure 2A) and the bursting models segmented by the rising phase of the slow wave (Figure 2C,D). In the absence of clear subpopulations arising from model clustering, an effort was made to achieve optimal resolution within the conductance space by avoiding large differences in group size. For activity metrics without clear subpopulations, shifting the arbitrarily chosen population boundaries shown in Figure 2 did not drastically change the correlations found. Additional metrics were used to partition the periodic spiking and bursting models, such as spike height and the number of spikes per burst, respectively. However, these metrics resulted in a lower overall success rate for finding correlations and were therefore not reported here.

The segmentation scheme described above investigates the relationship between correlations and single activity characteristics. We also looked for correlations in model populations based on multiple activity characteristics. To do this, we constructed model populations based on subsets of the pacemaker activity criteria previously described, including characteristics of the “slow wave” voltage oscillation underlying bursts in STG pacemaker neurons [28]. Briefly, the slow wave is the membrane potential of a bursting model after spikes have been subtracted. The peak of the slow wave was approximated by the last maximum in a burst, and the slow wave amplitude is then the difference between this maximum and the between-burst minimum. The ranges of activity characteristics used are based on experimental data from the spontaneously bursting pacemaker kernel of the STG, which consists of one anterior burster (AB) neuron electrically coupled to two PD neurons. The complete dataset, including the descriptive statistics used to classify models, is available online as a supplemental file (datasets S1 and S2).

### Conductance plots

**Figure 3:**
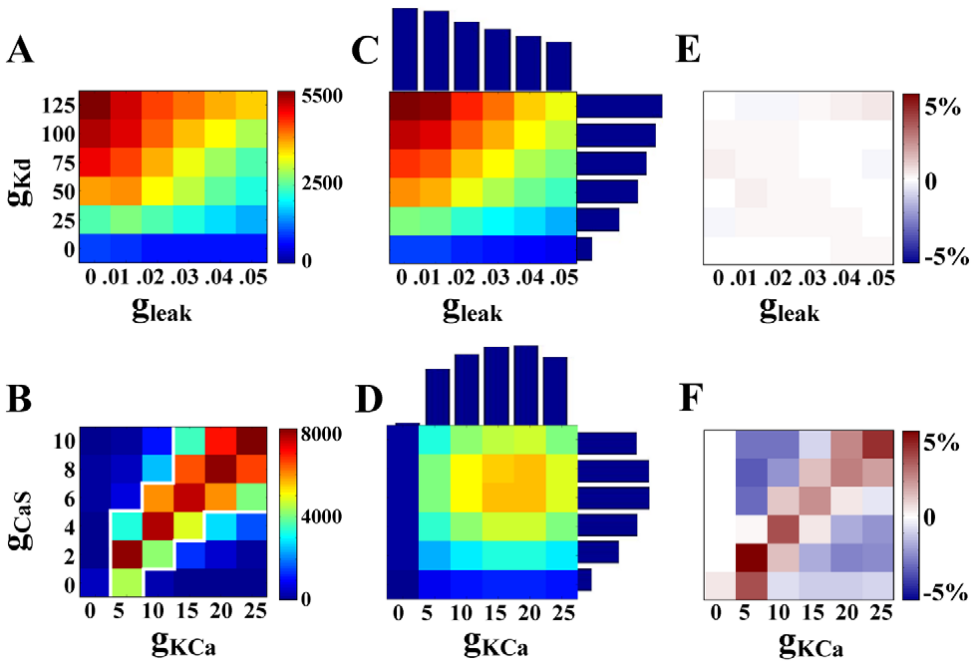
Ramp-type conductance relationships can be fully explained by the independence assumption, whereas linear correlations cannot. (A) The activity type “periodic bursters with duty cycle 0.1–0.2” tends toward high values of g_Kd_ and low values of g_leak_ (χ^2^ = 253, ρ = 0.006). Colors represent the number of models on each grid point. All maximal conductance units are mS/cm^2^. For each plot, no assumptions are made about the values of the other six conductances. (B) The same activity type shows a linear relationship between the CaS and KCa conductances (χ^2^ = 72,330, ρ = 0.627). The white outline encases correlation boundaries as used for creating a correlation-based population, see methods. (C,D) Data generated under the independence assumption. 1-D histograms protrude from the axis of the conductance they represent. The resulting independence matrix was generated by multiplying the two 1-D histograms, then scaling by total number of models in this activity group (see Methods). Color scale is the same in A and C and in B and D, respectively. (E,F) Difference matrix. The independence matrix (C,D) was subtracted from the actual data (A,B). Colors in (E,F) represent percent difference between independence matrix and actual data at each grid point.

After model neurons were sorted based on intrinsic activity, conductance plots were generated for each activity subsection (Figure 1). As shown in Figure 3, these plots graph the value of one conductance parameter versus another, for a particular activity subsection. Each model in the activity subsection falls on one grid point on this plot, positioned by the maximal conductance parameters it was assigned for the pair in question. The other 6 conductance parameters were not constrained when plotting.

### Analysis of conductance relationships

The grid structure used to generate simulation points for this database creates a conductance space wherein all conductance values are initially equally represented. After segmentation of the database, this is no longer the case and each activity type has a unique distribution of each conductance parameter. Bursting models, for example, have a higher average value of g_KCa_ than spiking models. These non-standard one-dimensional (1D) conductance distributions violate the assumptions of parametric tests and significance testing. Instead, we used two non-parametric tests with simple cutoff values to define correlations. For each activity subsection: if the value of one conductance was determined to be dependent on the value of another (by a chi-squared test of independence χ^2^>500), and this dependence was confirmed to have a linear trend (by a Spearman's rank correlation |ρ|>0.2), the relationship was considered a correlation. Note that, in the absence of significance testing, these definitions are not dependent on the number of observations (models in each population). This was chosen purposefully because, due to the sparse sampling of the conductance space, populations with very few models may not contain enough information to reliably identify correlations. Cutoff values independent of the number of observations ensures that those correlations reported are strong in all cases.

In addition to the described statistics, a simple visual check for a linear dependence was performed as follows. Assuming independence between conductances, it is possible to generate the expected two-dimensional (2D) conductance distribution for a particular activity type by multiplying normalized single-conductance histograms for that activity type (hereafter referred to as an independence matrix). By looking at the deviations in the actual data from this independent assumption, a linear dependence or lack thereof is more clearly visible than when looking at the correlation plots alone (Figure 3E,F). We chose to use this simple, graphical representation of dependence as a visual check to distinguish correlations from conductance relationships that can be explained by independently varying parameters. Specifically, the independence matrix was created by multiplying two conductance histograms for a particular activity type (units in % of models of that activity type). Then, this independence plot was subtracted from the actual data (units in % of models of that activity type). Note that the resulting plot of percent difference (hereafter referred to as a difference matrix, shown in Figure 3E,F) is distinct from, but complementary to, the chi-squared test of independence.

### Correlation-based populations

Defined correlations were used to generate populations of model neurons that fit within those correlation boundaries. The shape of each correlation was defined by setting a threshold of 3% (models/all models in the activity type, the latter hereafter referred to as type_i_) per grid point of the correlation plot raw data (see the white outline in Figure 3B for an example). The entire database, regardless of activity type, was scanned for models that fell within the boundaries of one defined correlation or a combination of correlations. We call the resulting “correlation-based” population cb_i_.

If a correlation is successful in restricting activity to a particular type, it is expected that a large number of models in the correlation-based population would be of that type. Specifically, we would expect that there would be a greater percentage of type_i_ models in cb_i_ when compared to the original database. We therefore calculated a statistic we termed success percentage (%Success), by dividing the number of models in cb_i_ that are of type_i_ by the total number of models in cb_i_ (%Success_Correlation-based_). The percent of type_i_ models in the original database (%Success_Original_) was also calculated (see percentages shown in Figure 1). The two were compared as follows:
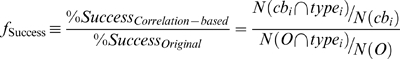
where O represents the original database, N denotes a function returning the number of models in the parameter population, and O∩type_i_ should be read as “the intersection of the original database and the set of models of type_i_”. The success factor (*f*_Success_) is therefore a multiplicative factor by which implementing a correlation increases or decreases the likelihood of a particular activity type. An increase in % success for the correlation-based population versus the original database (*f*_Success_>1) indicates a correlation that is useful in supporting a desired activity type.

As a control, *f*_Success_ was also calculated for a randomly distributed conductance plot applied to a random conductance pair. For the case of multiple correlations, the control case employed the same number of randomly generated conductance plots as there are defined correlations for that activity type. As further verification of our methods, we also applied an ideal linear correlation shape to randomly selected conductance pairs and activity types, and found no unexpected increases in *f*_Success_ (data not shown).

## Results

### Conductance relationship types

**Figure 4:**
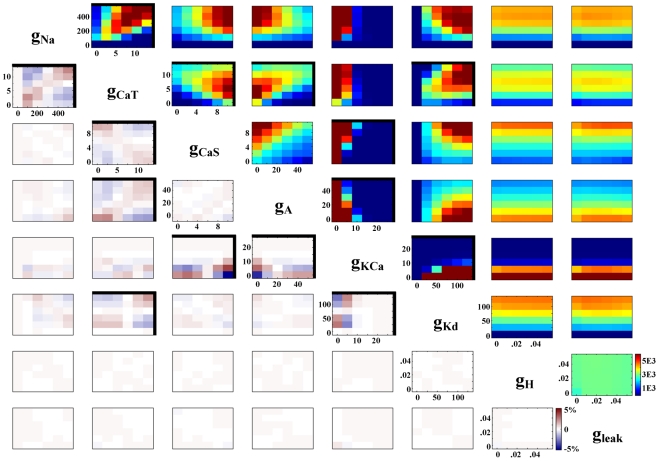
All possible pair-wise conductance combinations for the group of spiking models with frequency between 50 and 75 Hz (upper right). Color scheme represents the number of models. The lower left half of the plot contains the difference matrices for this activity type. The blue/red color scheme represents percent difference between data and the independence assumption (see Methods). For all plots, a bold border indicates a linear dependence according to our criteria (χ^2^>500 and |ρ|>0.2).

A model neuron database partitioned by activity type was screened for pair-wise conductance relationships, to elucidate the possible functional role of linear conductance relationships seen in experiments [6], [8], [9]. However, linear dependence was not the only relationship type seen in this database. A wide variety of nonlinear relationship types were also seen (for examples, the correlation plots of activity type “spiking models 50–75 Hz” are used, Figure 4). Many conductance pairs were clearly independent of one another; examples include completely flat distributions (g_H_ and g_leak_, Figure 4) or correlation plots with a striped appearance (g_Na_ and g_H_, Figure 4). In these cases, one or both of the conductances involved has a flat 1D distribution, and therefore the activity type in question does not require a particular value or range of values of that conductance. Another type of relationship commonly seen was the “ramp” type. These conductance plots had a high concentration of models in one corner, few models in the opposite corner, and a gradient in between. Intuitively, this would suggest dependence. On the contrary, many of these apparent relationships could be explained by independent variation of conductances. For example, the g_CaS_ and g_A_ conductance plot shown in Figure 4 is one example of a ramp type relationship where the corresponding chi-squared statistic was low, and the percent-difference plot between actual data and the independence assumption (difference matrix) shows no interesting trend. Indeed, this relationship appears to be fully explained by independent variation of parameters, as many of the ramp-type relationships we saw were (Figure 3A,C). Finally, both positive (g_Na_ and g_CaT_, Figure 4) and negative (g_CaT_ and g_CaS_, Figure 4) linear correlations were seen and will be described in detail.

### Efficacy of linear dependence statistics

**Figure 5:**
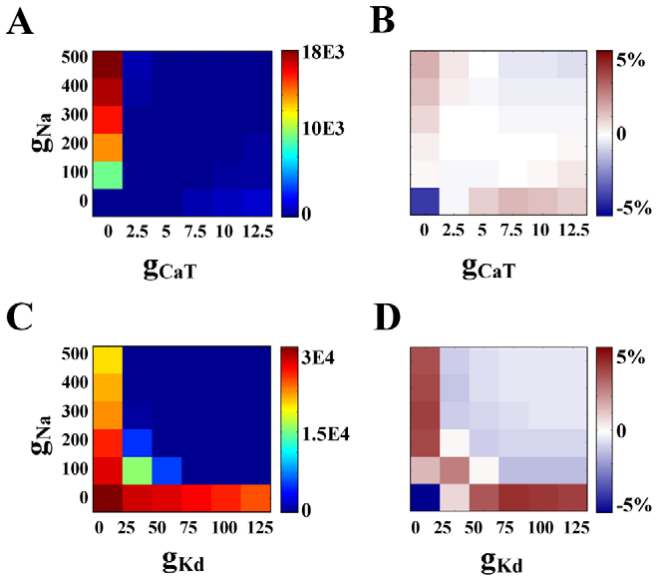
Eleven conductance relationships met the cutoff criteria for correlation, but did not have a convincing linear trend upon visual inspection. These false-positives were not included in the correlation totals. (A) Example of plot which met criteria for correlation, but not linearity. From group “spikers <10 Hz”. (B) Difference plot for relationship in A. (C) Another example. From group “one-spike bursters”. (D) Difference plot for relationship in C.

**Figure 6:**
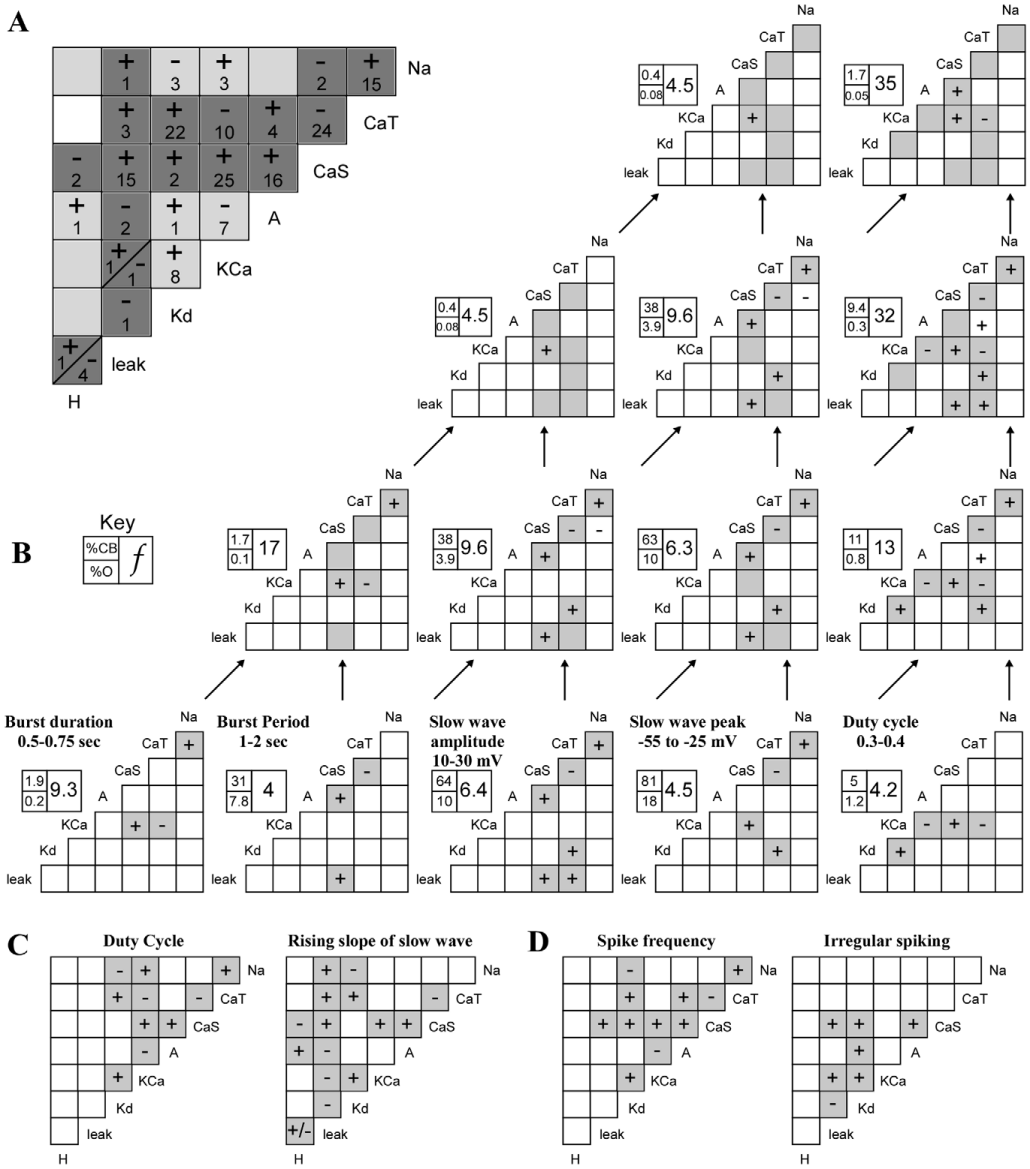
Each activity type utilizes a unique combination of conductance correlations. For all plots, a plus sign (+) indicates that a linear relationship with a positive slope was found for this conductance pair. Likewise, a minus sign (−) indicates a linear relationship with a negative slope. (A) Summary of entire database. If a correlation was seen, in any activity subsection investigated, it is included here. Conductance pairs shown in dark grey are those that have not yet been observed experimentally, whereas those highlighted with light grey have been found in experiments on STG neurons [8], [9]. Two negative relationships reported here (g_Na_ vs. g_Kd_ and g_A_ vs. g_KCa_) were found to be positive relationships when investigated experimentally by Schulz et al [9]. However, there is also agreement with experimental results, such as a positive correlation (g_A_ vs. g_H_) found by Khorkova and Golowasch [8]. Numbers indicate how many activity types showed a particular conductance correlation. (B) Example of how restricting multiple aspects of activity type may influence the appearance of correlations. The bottom row shows correlations for populations based solely on one of the previously published pacemaker criteria [28]. Arrows indicate populations based on combinations of these criteria. Shading here is used only to show that a correlation was present in a “parent” population; if a plus or minus sign is absent then no correlation was observed. %CB is %Success for a correlation-based population, %O is %Success for the original database, and f is the ratio of the two, or f_Success_. See Methods. (C) Periodic bursting models were either segmented by duty cycle, or the slope of the rising phase of the slow wave. The correlations of all activity subsections created for each schema are summarized here. Shading is used for contrast only. (D) All correlation types seen in any of the spike frequency defined activity types and all correlation types seen in the irregular spiking group.

The statistical criteria used to define correlations were chosen with the goal of distinguishing those conductance pairs linearly dependent on one another. To verify the statistics, each plot and its corresponding difference matrix were checked for visual confirmation of a linear trend (Figure 4). In a small number of cases (16/174) we found that a plot would fit the statistical criteria for linear dependence but linearity was not evident. This was most often an edge effect; for instance, a relationship wherein one or both conductances were zero for a majority of the models (Figure 5). Notably, most false positives were due to low average values of g_KCa_ or g_CaT_ or both, and were found in activity types such as silent and fast spiking models. False-positives, though they met the statistical criteria, were not considered correlations (see Supplemental files, Table S2). Statistical criteria were chosen based on minimization of false-positive results. Excluding those cases discussed above, our statistical criteria identified 174 pair-wise linear correlations out of a possible 1316 conductance pair combinations in 47 model sub-populations (see Figure 6B and Supplemental files Table S1).

**Figure 7:**
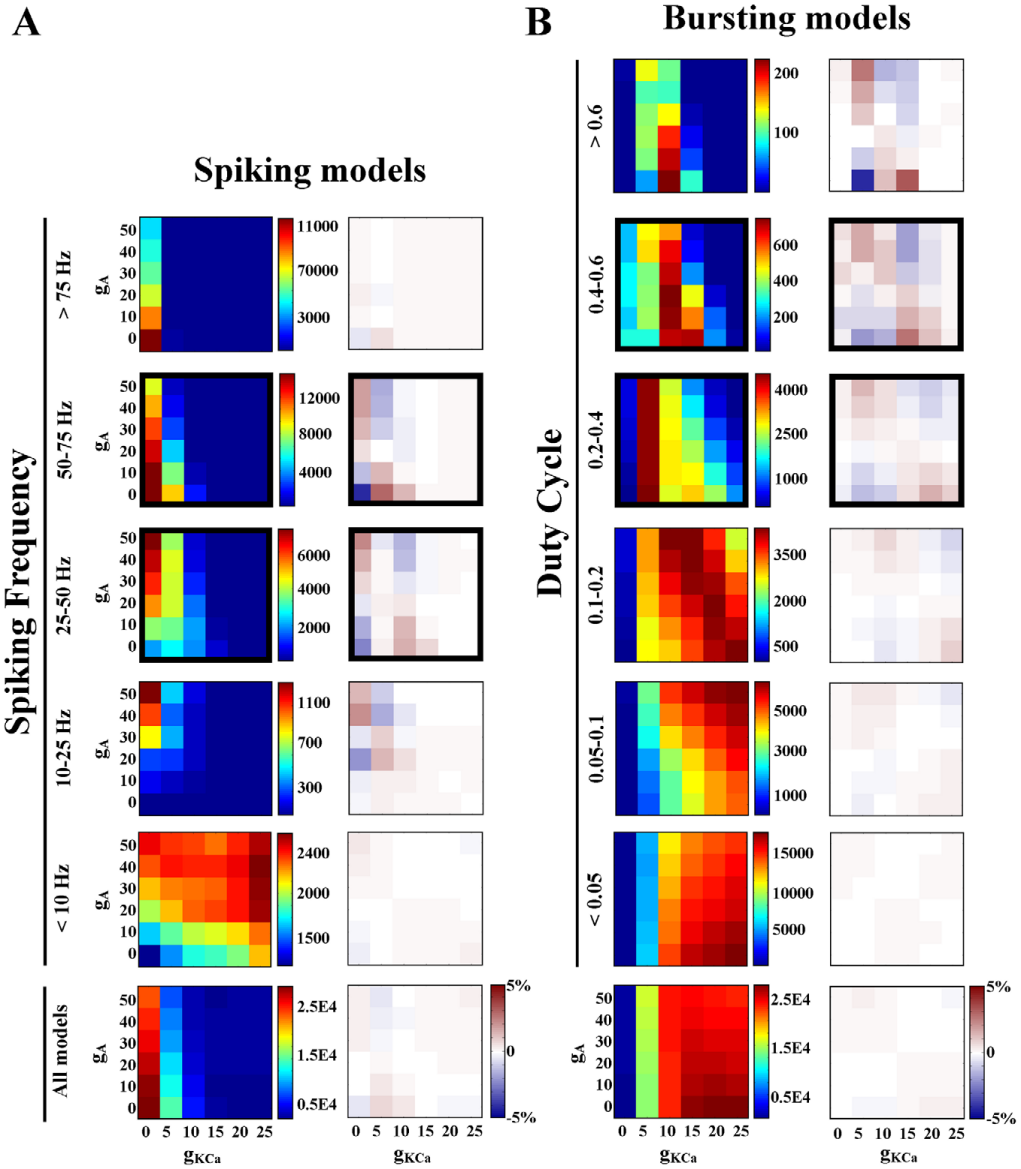
Values of g_A_ and g_KCa_ are correlated for spiking models with a frequency between 25 and 75 Hz and bursting models with duty cycle between 0.2 and 0.6. Spiking models are represented by the leftmost two columns (A), and bursting models are shown to the right (B). Frequency or duty cycle range is specified to the left of each pair of plots, excluding the bottom row which represents all spiking or bursting models. Bold black borders indicate a correlation (χ^2^>500 and |ρ|>0.2). The g_A_ and g_KCa_ relationship in bursting models with duty cycle >0.6 (top right) was considered a false negative result (χ^2^ = 262, ρ = −0.33).

We did not tally the relationships that appeared linear but did not meet the cutoff criteria (apparent false-negatives) because they tended to occur only in activity subsections with relatively low numbers of models. A property of the chi-squared test of independence, that relationships with more data points will more easily reach a cutoff value, was intentionally used to compensate for the sparse sampling of the conductance space. One example of an apparent false negative is shown in Figure 7. The activity type “bursting models with a duty cycle greater than 0.6” appears to have a linear dependence between g_A_ and g_KCa_; however, the chi-squared statistic is less than 500 for this plot. A possible solution to avoid excluding this apparent correlation would be to use a chi-squared statistic that is scaled by the total number of models in the population, because this group contains only 1776 models compared to an average of 100,000 (Table S1). However, that approach would exclude other, possibly more reliable, relationships. For example, a scaled cutoff would exclude the g_A_ and g_KCa_ correlation from the activity type “bursting models with a duty cycle between 0.2 and 0.4” which appears very similar to the ‘false negative’ discussed above (Figure 7). We chose to use the raw statistic (unscaled) because we found this result counter-intuitive. A relationship found in a population with a large number of models should be more reliable than an equivalent relationship found in a population with very few models, especially on a sparse grid. This is not to say that relationships fitting our statistical criteria were not apparent in populations with low numbers of models. On the contrary, we found several correlations in this and other populations with relatively few models (Table S1).

### Model populations based on activity characteristics

When model populations were defined by a single activity characteristic, a correlation was more likely to appear in a group with a narrow range of that characteristic (Figure 1). For example, the group “all periodic spikers” had no correlations; however, when this group was segmented by spike frequency every subsection had at least one correlation. Similarly, the group “all periodic bursters” demonstrated no correlations, whereas all of its subsections partitioned by duty cycle did. Although the categories without correlations each included over 200,000 model neurons (see Figure 1 and Supplemental files, Table S1), this phenomenon was not linked to the size of the activity subsection. For example, the group “bursters with duty cycle <0.05” contains a large number of models (392,398) and has three correlations. It is reasonable then to assume that correlations arise by virtue of the narrowly defined activity type of a group rather than simply the number of models in a group.

**Figure 8:**
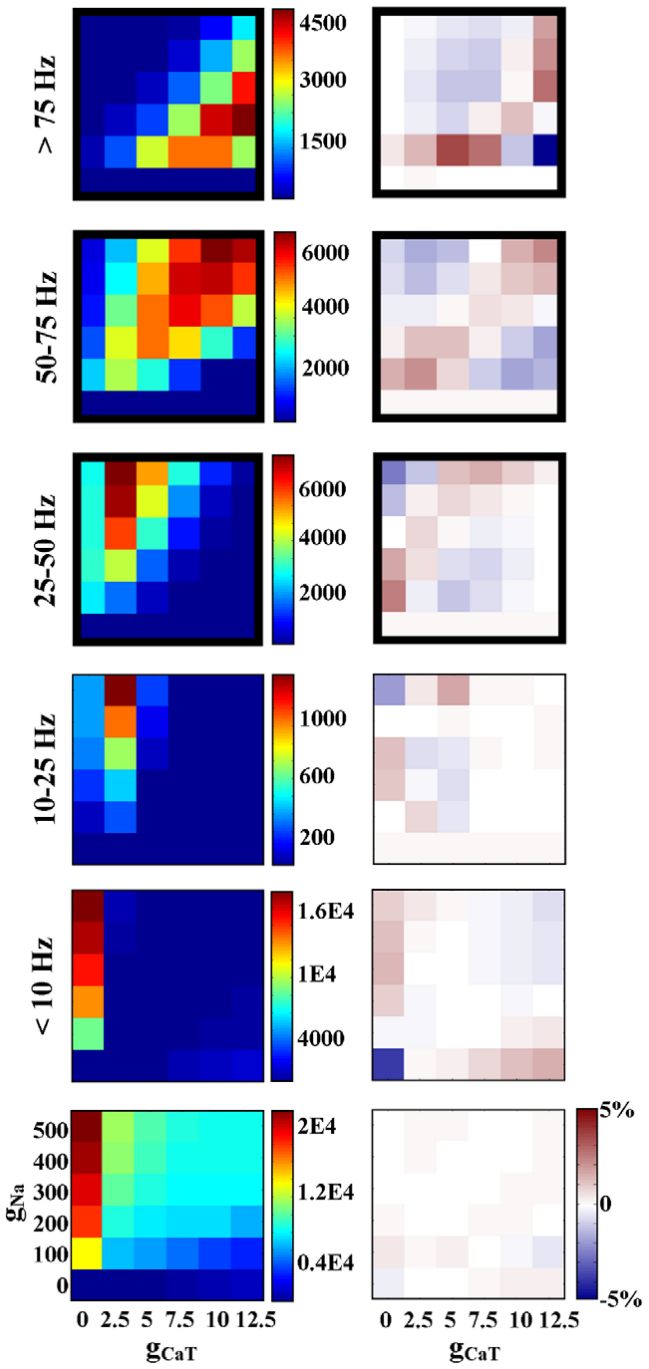
Values of g_Na_ and g_CaT_ are correlated for spiking models with a frequency greater than 25 Hz. Bold black borders indicate a correlation (χ^2^>500 and |ρ|>0.2).

Very few conductance pairs were seen to demonstrate a positive correlation in one activity type and a negative correlation in others (Figure 6A). Thirteen conductance pairs exhibited only positive correlations, eight conductance pairs exhibited only negative correlations, and two conductance pairs had both relationship types. We found no correlations for the remaining five possible conductance pairs. As shown in Figure 7, the relationship between g_A_ and g_KCa_ is one example of a negative correlation. This conductance pair was negatively correlated for spiking models with a frequency of 25–75 Hz (subsections 25–50 Hz and 50–75 Hz) and bursting models with a duty cycle between 0.2 and 0.6 (subsections 0.2–0.4, and 0.4–0.6) (Figure 7). Interestingly, though all of the correlations for the g_A_/g_KCa_ pair are negative, they appear to have a slightly different slope in each case. This is only one example of slope differences seen between activity types for a single conductance pair, though there are also cases of correlations with the same slope in several activity groups. In the latter case, correlations in different activity groups generally inhabit different areas of the conductance space (Figure 8).

There was a strong presence of calcium conductance correlations in this database (Figure 6A). In the model sub-populations we investigated, there were 140 correlations involving one or both calcium conductances, compared to only 34 that did not involve either calcium conductance. All conductances showed correlations with at least one of the calcium conductances. The g_KCa_ and g_CaS_ conductance pair had the highest number of correlations and was strongly correlated in several activity types.

In contrast with populations based on a single criterion, there was no straightforward trend in the appearance or disappearance of correlations when multiple activity criteria were used to generate a population. Individually, each population based on a single pacemaker criterion has a unique set of correlations (Figure 6B, bottom row). When criteria are combined, however, correlations appear or disappear with a seeming disregard to those seen in the “parent” populations. For example, starting with the population based on slow wave amplitude (SWA) between 10 and 30 mV, adding the slow wave peak (SWP) range as a criterion leads to a loss of the g_CaT_ vs. g_leak_ correlation. Adding duty cycle as a criterion brings back the g_CaT_ vs. g_leak_ correlation, even though the duty cycle parent population does not use it. Adding burst period again leads to loss of that correlation. There is no path from bottom to top in Figure 6 along which the number of correlations increases steadily with an increase in criteria. When all 5 criteria were used, no correlations were found, though this may be influenced by the small number of models that fit all 5 criteria (56).

### Correlation-based populations

**Figure 9:**
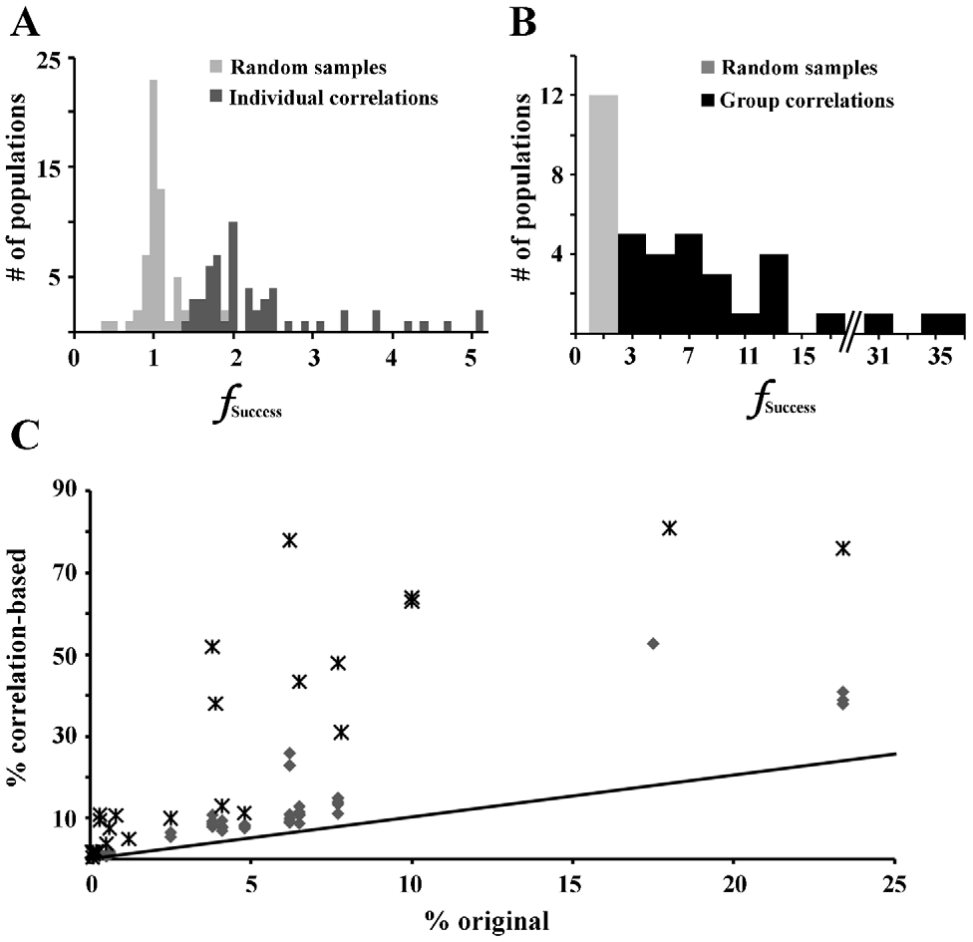
Correlation-based populations increased the percentage of models with a desired activity type. (A) Implementation of correlations individually had a modest, but always positive, effect on the percentage of models with the desired intrinsic activity type (%Success), contrary to the random-sample controls. Histograms are stacked in the rare case of overlap. Bin size is 0.1%. (B) Implementation of all correlations seen in an activity type increased %Success by as much as 37 fold. Bin size is 1%. (C) The percentage of a particular activity type in the original database (% original) is plotted against the percentage of models of that activity type in a correlation-based population (% correlation-based). Both single-correlation based populations (grey triangles) and multiple-correlation based populations (black stars) are shown. Unity line is shown for scale.

Finally, defined correlations were used to create correlation-based sub-populations of models as described in the Methods section. A correlation-based population was, in all cases, found to have a larger percentage of models of the desired activity type than the original database. We found a 2.3 fold increase for individual correlations, on average (σ = 0.9). Individual correlations ranged from having a small positive effect on success rate to increasing it 5 fold (Figure 9). This is in contrast to the control condition of random conductance relationships, in which decreases in success were as likely as increases. On average, there was no difference between the control condition and the original database (average *f*_Success_ = 0.98, σ = 0.2).The difference between control and correlation-based populations was even larger when multiple correlations were used to generate each population. When the set of all correlations for an activity type was used to create a population of models, the percent success increased 10 fold on average (Table S3). In contrast, the average *f*_Success_ for the control case with multiple random conductance distributions was below 1 (μ = 0.89, σ = 0.17).

## Discussion

Our results illuminate the functional benefit of linear conductance correlations for maintaining activity type. When a population of models was gathered based on adherence to a correlation rule, there was always an increase in a particular feature of activity. In some cases, this increase was admittedly modest, particularly when the population was based on a single correlation. However, there were several cases in which the constraint on activity type was impressive (Figure 9). In one case, imposing a single correlation caused an increase in the desired activity type from 18% (in the original database) to 53% (in the new, correlation-based population). Another population increased its percentage of a single activity type by 72% when multiple correlations were used. Put another way, the combined correlation-based populations demonstrated up to 81% of a single activity type. We find this to be a notable contribution to robustness of activity.

This model implicates two types of correlations not previously seen in experiments. As of this writing there have been no published calcium conductance correlations. We have shown that calcium correlations are plausible, and if implemented by a cell, would assist maintenance of a wide range of activity types. This is also true of correlations with a negative slope, which have not yet been seen experimentally. Thirty percent of all correlations seen (56/174) were negative, spanning 10 out of the 23 conductance pairs with correlations in this study (see Supplemental files, Table S1). Though the mechanisms underlying biological correlations are uncertain, and not represented in the details of our model, our results suggest that negative correlations can be just as useful as positive ones for maintaining cellular activity.

Negative correlations have been hypothesized for conductances that may compensate for one another. For example, a study of the solution space of a multi-compartment cerebellar Purkinje cell model reports negative correlations between several pairs of conductances, including two calcium conductances that appear to be anti-correlated so as to preserve the total calcium influx into the cell [32]. When fitting model neuron parameters to reproduce specific experimentally recorded neuronal voltage traces, such functionally compensatory conductances can lead to irreducible model parameter uncertainty [33]. An analysis of the correlation between the two potassium conductances g_KCa_ and g_A_ shows this type of relationship in our model. The Ca^2+^-dependent K^+^ current is often associated with bursting cells, and contributes in our model to determining burst length. Keeping all other conductances constant in a bursting model neuron, a higher value of g_KCa_ will correspond to a shorter burst length [34]. The A current is also a K^+^ current, though it is not calcium dependent and it acts on a much faster time scale. Keeping all other conductances constant in a bursting cell, a higher value of g_A_ will result in a slower spike rate, or fewer spikes per burst [35]. The effects of these conductances appear to sum for bursting neurons with a duty cycle between 0.2–0.6 (Figure 7). To maintain duty cycle in this range, a model needs a minimal amount of one or both conductances to counter-balance depolarizing (inward) currents. The exception to this rule is a small population (∼300 models) of elliptic bursters in the 0.4–0.6 duty cycle group. These models all have the same low value of g_Na_ (100 mS/cm^2^), which may allow the absence of both g_KCa_ and g_A_. Interestingly, bursting models with a low duty cycle show no dependence between g_A_ and g_KCa_.

A second type of negative correlation was seen between g_Na_ and g_Kd_. Initially, this negative correlation was surprising. Both conductances are involved in generation of the action potential and would therefore be expected to require balance via a positive correlation. Instead, only negative correlations were seen, and they appeared in two activity subsections: spiking models with frequency greater than 75 Hz, and bursting neurons with a duty cycle less than 0.05. We found, based on where these correlations appeared, that a negative relationship between these two conductances was useful for maintaining very fast spiking. If a model in either of these activity groups was adjusted to make both conductances very low, then the model became silent. If both conductances were very high, the spike would broaden and the spiking frequency would decrease. It is interesting to note that both the conductance pairs discussed so far have been found to be positively correlated experimentally in stomatogastric neurons [9]. However, the activity types found to have these correlations in the database are not typical of stomatogastric intrinsic activity types [1], [36], [37] and thus should not be compared directly to experiment.

There are several reasons why directly relating our results to the published experimental data is a challenge. First, conductance is not directly measured in experiments. Mapping conductance (used in the database) to mRNA copy number or current density involves several tenuous assumptions. For example, conversion between mRNA copy number and conductance value has been studied for three conductance types in the STG, of which only two were found to relate linearly [3], [38]. Therefore, it cannot be assumed that the other 5 conductances types used in this model have a linear relationship between mRNA copy number and conductance value. A larger problem is the choice of comparison: which activity characteristics should be assigned to a biological cell in a particular experiment? Our analysis of activity type of the model did not consider the effects of current injection or neuromodulation, and therefore should be compared to intrinsic activity of the biological cells. Unfortunately, there are conflicting accounts of the intrinsic activity of cells in the stomatogastric ganglion. While they have reliable and identifiable activity types in the intact circuit, isolating a cell to measure intrinsic activity often involves direct harm to its own processes or neighboring cells. The method of isolation may influence activity in unknown ways. For example, when the PD neuron is cut off from all synaptic input and neuromodulation it can be spontaneously silent [39], spike tonically [39], [40], burst with a periodic rhythm [39] or burst irregularly [40]. The one exception in the STG is the AB neuron, which is an intrinsic burster and has the same intrinsic and network behavior. Unfortunately, no experiments have been done examining conductance correlations in the AB neuron. For these reasons, we chose to avoid comparing our results for a particular activity type with the experimental data for a single cell type.

Even so, there are general comparisons that can be made between our results and experiment. For instance, we found differences in slope for the same correlation in different activity types (Figure 7). This is reminiscent of the cell-type specific correlations found by Schulz et al.; the correlation slope for a single conductance pair was different in each cell type [9]. In addition, we saw correlations which maintained slope but differed between activity types with regard to intercept (Figure 8). We can predict from this result that the general location within the conductance space is also important for determining activity, which has been suggested before [23]. Furthermore, each identified cell type studied by Schulz et al. had a unique combination of correlated conductance pairs, similar to our results (see Supplemental files, Table S1). This suggests that not only the slope and location of correlations, but the combinations in which they are used, are important for definition of cellular identity. This view is bolstered by our results with correlation-based populations. We showed that combinations of correlations, drawn from a particular activity type, generated much larger increases in percent success compared to individual correlations or controls (Figure 9).

Another important comparison to experiment involves the hyperpolarization-activated inward conductance, g_H_. Correlations involving this conductance were only found when the slope of the slow wave was used to identify activity type (Figure 6C). We found this result to be particularly interesting because correlations involving g_H_ have been found repeatedly in experiments in the stomatogastric ganglion [6], [7], [8], [9]. In experiments on STG PD and LP cells, both the slope of the slow wave and the ratio of I_A_ to I_H_ were shown to be conserved after injection of shal mRNA [6].This implies that the correlation between the values of I_A_ and I_H_ may have something to do with maintaining the shape of the slow wave. In these experiments, the average slope of the rising phase of the slow wave was found to range, roughly, from 0.02–0.08 mV/ms. We found this correlation in a slightly higher range when looking at the central 50% of the rise phase (>0.1, see supplemental Table S1). Though these results are slightly different, both correlations were positive and both suggest that I_H_ is involved in maintaining a specific activity characteristic: inter-burst dynamics. Interestingly, I_H_ was not correlated in any other activity types. We hypothesize that this is because the other activity types are defined more by conductances which are active during the depolarized phase of the spike or burst. If this is the case, it would follow that correlations involving I_H_ would be more prevalent if response to hyperpolarizing current injection were used as a metric for defining activity type. For cells dependent on inhibitory network connections for a bursting phenotype, such as LP and pyloric (PY) neurons in the STG, this may be the case and should be an interesting area of further research.

Recently, Taylor et al published a study of a population of LP neuron models, constrained by 9 activity characteristics, in which no strong linear correlations were found. We report a similar result in that the usefulness of correlations for supporting activity characteristics did not scale up as we expected. When multiple biologically-realistic activity criteria were used to generate a population of models, the appearance and disappearance of correlations was not easily explained by the correlations found in the “parent” single-criteria populations. This suggests that the correlations which would be helpful for each feature individually might interact in complex ways, inhibiting or enhancing the influence of the other, when multiple activity characteristics are imposed on a group. In our case, a likely explanation for these results lies in the complexity of the conductance space. The conductance plots we analyzed were 2D approximations of an 8-dimensional space. This means that a perfect linear correlation between two parameters will appear as such. However, dependencies between multiple parameters will form subspaces within the overall conductance space that may, or may not, be detectable in a flattened 2D analysis. Furthermore, the perfect population definition for one conductance pair may not be ideal for another conductance pair, resulting in the occasional conductance plot that contains two local dependency rules (see Figure 5A for a possible example). Finally, the scale of analysis is important. We had the opportunity to examine raw experimental data from correlations published by Schulz et al. [9]. It has been previously shown that every STG cell type has a unique range of conductance values [3], [38]. By binning the experimental data based on the conductance ranges of each cell type, it was apparent that bin size could contribute to the appearance of correlations (analysis not shown). For example, when binning conductance measurements from the gastric mill (GM) cell type based on the range of conductance values present in the PD cell type, otherwise apparent correlations are lost. Our model database is limited in this way, due to the grid structure of the conductance space, which can be interpreted as a type of binning. Combined, this complex behavior can easily give rise to situations in which correlations appear, or disappear, as the population of models is further reduced. Though this result highlights the inability of our analysis to capture all possible linear dependencies between conductances, it is important to note that it does not shed doubt on the correlations we did find.

Though our set of apparent correlations should not be considered an inclusive list for these activity types, the correlations found in our database are useful for considering the possible effects of conductance relationships on activity. We argue that the presence of a large number of defined conductance relationships lends to the validity of the database as a tool for investigating correlation utility. With this tool, we have shown that linear conductance correlations can shape neuronal activity. Furthermore, we made specific predictions about the presence of negative or g_Ca_ correlations and situations in which they might be useful.

## Supporting Information

Table S1 Linear conductance relationships (χ ^2>500 and |ρ|>0.2) by activity type. Any correlation with a |ρ|>0.4 is shown in bold, and any correlation with a |ρ|>0.6 is shown in red.
(0.33 MB RTF)

Table S2 Conductance relationships that fit statistical criteria for correlations, but do not appear to have a linear relationship.
(0.09 MB RTF)

Table S3 Percent success increased an average of 10 times the original value when model populations were defined by correlations. Correlation-based populations were generated using the complete set of correlations that define that activity type in the original database (See Table S1).
(0.07 MB RTF)

Dataset S1 Model database part 1. One-half of the model database. The file ‘README.txt’ describes the layout of the ‘databasePt1.txt’ file.
(7.50 MB ZIP)

Dataset S2 Model database part 2. Part 2 of the model database. The file ‘README.txt’ (included in the Dataset S1 zip file) describes the layout of the ‘databasePt2.txt’ file.
(9.22 MB ZIP)

## References

[1] Harris-Warrick RM, Marder E, Selverston AI, Moulins M (1992). Dynamic Biological Networks: The Stomatogastric Nervous System.

[2] Prinz AA, Bucher D, Marder E (2004). Similar network activity from disparate circuit parameters.. Nat Neurosci.

[3] Schulz DJ, Goaillard JM, Marder E (2006). Variable channel expression in identified single and electrically coupled neurons in different animals.. Nat Neurosci.

[4] Marder E, Thirumalai V (2002). Cellular, synaptic and network effects of neuromodulation.. Neural Netw.

[5] Marder E, Prinz AA (2002). Modeling stability in neuron and network function: the role of activity in homeostasis.. Bioessays.

[6] MacLean JN, Zhang Y, Goeritz ML, Casey R, Oliva R (2005). Activity-independent coregulation of I-A and I-h in rhythmically active neurons.. J Neurophysiol.

[7] MacLean JN, Zhang Y, Johnson BR, Harris-Warrick RM (2003). Activity-independent homeostasis in rhythmically active neurons.. Neuron.

[8] Khorkova O, Golowasch J (2007). Neuromodulators, not activity, control coordinated expression of ionic currents.. J Neurosci.

[9] Schulz DJ, Goaillard JM, Marder EE (2007). Quantitative expression profiling of identified neurons reveals cell-specific constraints on highly variable levels of gene expression.. Proc Natl Acad Sci U S A.

[10] Peng IF, Wu CF (2007). Drosophila cacophony channels: A major mediator of neuronal Ca2+ currents and a trigger for K+ channel homeostatic regulation.. J Neurosci.

[11] Linsdell P, Moody WJ (1994). Na+ channel mis-expression accelerates K+ channel development in embryonic xenopus-laevis skeletal-muscle.. J Physiol (Lond.).

[12] Vahasoyrinki M, Niven JE, Hardie RC, Weckstrom M, Juusola M (2006). Robustness of neural coding in Drosophila photoreceptors in the absence of slow delayed rectifier K+ channels.. Journal of Neuroscience.

[13] McAnelly ML, Zakon HH (2000). Coregulation of voltage-dependent kinetics of Na+ and K+ currents in electric organ.. J Neurosci.

[14] Colvis CM, Pollock JD, Goodman RH, Impey S, Dunn J (2005). Epigenetic mechanisms and gene networks in the nervous system.. J Neurosci.

[15] Gomez-Ospina N, Tsuruta F, Barreto-Chang O, Hu L, Dolmetsch R (2006). The C terminus of the L-type voltage-gated calcium channel Ca(V)1.2 encodes a transcription factor.. Cell.

[16] Zhang Y, Oliva R, Gisselmann G, Hatt H, Guckenheimer J (2003). Overexpression of a hyperpolarization-activated cation current (Ih) channel gene modifies the firing activity of identified motor neurons in a small neural network.. J Neurosci.

[17] Ouyang Q, Goeritz M, Harris-Warrick RM (2007). Panulirus interruptus I-h-channel gene PIIH: Modification of channel properties by alternative splicing and role in rhythmic activity.. J Neurophysiol.

[18] Baro DJ, Quinones L, Lanning CC, Harris-Warrick RM, Ruiz M (2001). Alternate splicing of the shal gene and the origin of I-A diversity among neurons in a dynamic motor network.. Neuroscience.

[19] Bito H, Deisseroth K, Tsien RW (1997). Ca2+-dependent regulation in neuronal gene expression.. Curr Opin Neurobiol.

[20] Fields RD, Lee PR, Cohen JE (2005). Temporal integration of intracellular Ca2+ signaling networks in regulating gene expression by action potentials.. Cell Calcium.

[21] Johnson BR, Kloppenburg P, Harris-Warrick RM (2003). Dopamine modulation of calcium currents in pyloric neurons of the lobster stomatogastric ganglion.. J Neurophysiol.

[22] Hooper SL, Marder E (1987). Modulation of the lobster pyloric rhythm by the peptide proctolin.. J Neurosci.

[23] Goldman MS, Golowasch J, Marder E, Abbott LF (2001). Global structure, robustness, and modulation of neuronal models.. J Neurosci.

[24] Taylor AL, Hickey TJ, Prinz AA, Marder E (2006). Structure and visualization of high-dimensional conductance spaces.. J Neurophysiol.

[25] Burdakov D (2005). Gain control by concerted changes in I-A and I-H conductances.. Neural Comput.

[26] Olypher AV, Calabrese RL (2007). Using constraints on neuronal activity to reveal compensatory changes in neuronal parameters.. J Neurophysiol.

[27] Taylor AL, Goaillard JM, Marder E (2009). How multiple conductances determine electrophysiological properties in a multicompartment model.. J Neurosci.

[28] Prinz AA, Billimoria CP, Marder E (2003). Alternative to hand-tuning conductance-based models: construction and analysis of databases of model neurons.. J Neurophysiol.

[29] Turrigiano G, LeMasson G, Marder E (1995). Selective regulation of current densities underlies spontaneous changes in the activity of cultured neurons.. J Neurosci.

[30] Huguenard JR, McCormick DA (1992). Simulation of the currents involved in rhythmic oscillations in thalamic relay neurons.. J Neurophysiol.

[31] Bucher D, Prinz AA, Marder E (2005). Animal-to-animal variability in motor pattern production in adults and during growth.. J Neurosci.

[32] Achard P, De Schutter E (2006). Complex parameter landscape for a complex neuron model.. PLoS Comput Biol.

[33] Huys QJ, Ahrens MB, Paninski L (2006). Efficient estimation of detailed single-neuron models.. J Neurophysiol.

[34] Sun Q, Dale N (1998). Developmental changes in expression of ion currents accompany maturation of locomotor pattern in frog tadpoles.. J Physiol.

[35] Tierney AJ, Harris-Warrick RM (1992). Physiological role of the transient potassium current in the pyloric circuit of the lobster stomatogastric ganglion.. J Neurophysiol.

[36] Heinzel HG, Selverston AI (1988). Gastric mill activity in the lobster. III. Effects of proctolin on the isolated central pattern generator.. J Neurophysiol.

[37] Bal T, Nagy F, Moulins M (1994). Muscarinic modulation of a pattern-generating network - control of neuronal properties.. J Neurosci.

[38] Baro DJ, Levini RM, Kim MT, Willms AR, Lanning CC (1997). Quantitative single-cell reverse transcription PCR demonstrates that A-current magnitude varies as a linear function of shal gene expression in identified stomatogastric neurons.. J Neurosci.

[39] Panchin YV, Arshavsky YI, Selverston A, Cleland TA (1993). Lobster stomatogastric neurons in primary culture .1. Basic characteristics.. J Neurophysiol.

[40] Bal T, Nagy F, Moulins M (1988). The pyloric central pattern generator in crustacea - A set of conditional neuronal oscillators.. J Comp Physiol A.

