# Supplementary material for: Conductance Ratios and Cellular Identity
Source: PLoS Comput Biol. 2010 Jul 1;6(7):e1000838. doi: 10.1371/journal.pcbi.1000838 (PMC2895636; doi:10.1371/journal.pcbi.1000838)
Supplement: Table S1 — Linear conductance relationships (χ ^2>500 and |ρ|>0.2) by activity type. Any correlation with a |ρ|>0.4 is shown in bold, and any correlation with a |ρ|>0.6 is shown in red. (0.33 MB RTF) [file pcbi.1000838.s001.rtf]

DATASET (# of models)	gleak	gH	gKd	gKCa	gA	gCaS	gCaT	gNa	
Silent (286400)	 	 	 	 	 	gCaT-	gCaS-	 	
BURSTING MODELS									
Non-periodic  bursters 
(55010)			gKCa+	gNa+
gCaS+
gKd+
gCaT-		gKCa+	gKCa-	gKCa+	
One-spike bursters 
(322679)	 	 	 	 	 	 	 	 	
All periodic bursters (712793)	 	 	 	 	 	 	 	 	
Periodic with	 	 	gCaT+	gNa+	 	 	gKd +	gKCa+	
duty cycle < 0.05 (392399)			gNa -					gKd -	
Periodic with	 	 	gCaT+	gCaS+	gCaS +	gKCa+	gKd +	gCaT+	
 0.05 <= duty cycle < 0.1 						gA+	gNa +		
 (129814)						gCaT-	gCaS-		
Periodic with	 	 	gCaT+	gCaS+	gCaS +	gKCa+	gKd +	gCaT+	
 0.1 <= duty cycle < 0.2				gCaT-		gA +	gNa+		
 (109781)				 		gCaT-	gCaS-		
 				 		 	gKCa-		
Periodic with	 	 	gKCa+	gKd+	gKCa-	gKCa+	gKCa-	 	
 0.2 <= duty cycle < 0.4				gCaS +					
 (68237)				gA-					
 				gCaT-					
Periodic with	 	 	gKCa+	gKd+	gKCa-	gKCa+	gKCa-	gKCa+	
 0.4 <= duty cycle < 0.6				gNa+		gCaT-	gCaS-		
 (10790)				gCaS +		 	 		
 				gA-		 	 		
 				gCaT-		 	 		
Periodic with	 	 	 	gCaS+	 	gKCa+	gNa+	gCaT+	
 0.6 <= duty cycle (1772)									
Periodic with average slope of the rise phase of the slow wave < 0.049  (306812)					gCaS+	gCaT-	gCaS-		
						gA +			
									
0.049 <= slope < 0.059 (54543)			gCaT+	gCaS+	gCaS+	gCaT-	gCaS-		
						gA +	gKd +		
						gKCa+			
0.059 <= slope < 0.072 (77577)	gCaS+
		gCaT+	gCaS+
	gCaS+
	gCaT-
gA +
gKCa+
gleak+	gCaS-
gKd +
		
									
									
0.072 <= slope (270500)	gCaS+
gH-	gleak-	gCaT+	gCaS+
		gKCa+
gleak+	gKd +
		
Periodic with first quarter slope of rise phase of slow wave < 0.04 (117978)									
0.04 <= slope < 0.06 (92608)	gH-	gleak-				gCaT-	gCaS-		
0.06 <= slope < 0.08 (115859)						gCaT-	gCaS-		
0.08 <= slope < 0.1 (153965)						gCaT-	gCaS-		
0.1 <= slope < 0.12 (133708)			gCaT+		gCaS+
	gCaT-
gA +	gCaS-
gKd +		
0.12 <= slope  (95313)	gCaT+		gCaT+	gCaS+		gKCa+
	gKd +
gleak+		
Periodic with central  slope of rise phase of slow wave < 0 (502)				gCaS+		gCaT-gKCa+	gCaS-		
0 <= slope < 0.04 (331386)	gCaS+				gCaS+	gCaT-
gA+
gleak+	gCaS-		
0.04 <= slope < 0.06 (91778)	gH+
gCaS+	gleak+	gCaT+	gCaS+	gCaS+	gA+
gKCa+
gleak+	gKd+		
0.06 <= slope < 0.08 (103874)	gCaS+		gCaT+	gCaS+	gCaS+	gA+
gKCa+
gleak+	gKd+		
0.08 <= slope < 0.1 (119006)	gH-
gCaS+
gA-	gleak-
gCaS-	gCaT+		gleak-	gleak+
gH-	gKd+		
0.1 <= slope  (62886)	gH-
gCaS+
gA-
gNa+
gKd-
gKCa-	gleak-
gCaS-
gA+	gCaT+
gNa-
gKCa+
gleak-	gKd+
gleak-	gleak-
gH+	gleak+
gH-	gKd+	gKd-
gleak+	
SPIKING MODELS	gleak	gH	gKd	gKCa	gA	gCaS	gCaT	gNa	
Non-periodic spikers
(8694)	gCaS+	 	gKCa+	gKd+	gKd+	gKd+	 	 	
	gKCa+		gA+	gleak+	gCaS+	gA+			
	gKd-		gleak-
gCaS+	 	 	gleak+			
All periodic spikers (294040)	 	 	 	 	 	 	 	 	
Periodic with	gCaS+	 	gCaS+	 	gCaS+	gKd+	 	 	
frequency < 10 Hz						gleak+			
 (79747)						gA+			
Periodic with	 	 	gCaT+	gCaS+	gCaT+	gKCa+	gKd+	 	
frequency 10-25 Hz						gCaT-	gA+		
 (5389)						 	gCaS- 		
Periodic with	 	 	gCaT+	gCaS+	gCaT+	gKCa+	gKd+	gCaT+	
frequency 25-50 Hz				gA-	gKCa-	gCaT-	gA+		
 (63465)				 	 	 	gNa+		
 				 	 	 	gCaS-		
Periodic with	 	 	gCaT+	gKd+	gCaT+	gKCa+	gKd+	gCaT+	
frequency 50-75 Hz			gKCa+	gCaS+	gKCa-	gCaT-	gA+		
 (103568)			 	gA-	 	 	gNa+ 		
 			 	 	 	 	gCaS-		
Periodic with	 	 	gNa-	 	 	 	gNa+	gCaT+ gKd-	
frequency > 75 Hz (41871)									
