# Supplementary material for: Conductance Ratios and Cellular Identity
Source: PLoS Comput Biol. 2010 Jul 1;6(7):e1000838. doi: 10.1371/journal.pcbi.1000838 (PMC2895636; doi:10.1371/journal.pcbi.1000838)
Supplement: Table S2 — Conductance relationships that fit statistical criteria for correlations, but do not appear to have a linear relationship. (0.09 MB RTF) [file pcbi.1000838.s002.rtf]

DATASET (# of models)	gleak	gH	gKd	gKCa	gA	gCaS	gCaT	gNa	
Silent (286400)	gH	gleak	gKCa	gKd	 	 	gKCa	 	
				gCaT					
BURSTING MODELS									
One-spike  bursters (322679)	 	 	gNa	 	 	 	 	gKd	
Periodic with first quarter slope of rise phase of slow wave < 0.02 (117978)	gCaT
gCaS
gA
gKCa
gKd
		gleak	gleak	gleak	gleak	gleak		
SPIKING MODELS									
Non-periodic spikers
(8694)	 	 	 	gCaT	 	 	gKCa	 	
All periodic spikers (294040)	 	 	 	gCaT	 	 	gKCa	 	
Periodic with	gCaT	 	gCaT	gCaT	 	gCaT	gNa	gCaT	
frequency < 10 Hz							gCaS		
 (79747)							gKCa		
 	 	 		 		 	gKd	 	
 	 	 	 	 	 	 	gleak	 	
