# Supplementary material for: Conductance Ratios and Cellular Identity
Source: PLoS Comput Biol. 2010 Jul 1;6(7):e1000838. doi: 10.1371/journal.pcbi.1000838 (PMC2895636; doi:10.1371/journal.pcbi.1000838)
Supplement: Table S3 — Percent success increased an average of 10 times the original value when model populations were defined by correlations. Correlation-based populations were generated using the complete set of correlations that define that activity type in the original database (See Table S1). (0.07 MB RTF) [file pcbi.1000838.s003.rtf]

		% Success		
Activity type	Original	Correlation	fSuccess	
Bursting	< 0.05	23.4	76.0	3.2	
	0.05-0.1	7.7	48.0	6.2	
	0.1-0.2	6.5	43.4	6.7	
	0.2-0.4	4.1	13.0	3.2	
	0.4-0.6	0.6	7.5	12.5	
	> 0.6	0.1	1.2	12.0	
Spiking	Irregular	0.5	3.7	7.4	
	< 10 Hz	4.8	11.2	2.3	
	10-25 Hz	0.3	10.9	36.3	
	25-50 Hz	3.8	51.9	13.7	
	50-75 Hz	6.2	78.0	12.6	
	> 75 Hz 	2.5	10.0	4.0	
	Average	5.0	29.6	10.0	
	St. dev.	6.4	28.3	9.3	
